# Current practice on the use of prophylactic drain after gastrectomy in Italy: the Abdominal Drain in Gastrectomy (ADiGe) survey

**DOI:** 10.1007/s13304-022-01397-0

**Published:** 2022-10-24

**Authors:** Valentina Mengardo, Jacopo Weindelmayer, Alessandro Veltri, Simone Giacopuzzi, Lorena Torroni, Giovanni de Manzoni, the Italian Gastric Cancer Research Group (GIRCG), the Polispecialistic Society of Young Surgeons (SPIGC), Ferdinando Agresta, Rita Alfieri, Sergio Alfieri, Nicola Antonacci, Gian Luca Baiocchi, Lapo Bencini, Maria Bencivenga, Michele Benedetti, Mattia Berselli, Alberto Biondi, Gabriella Teresa Capolupo, Fabio Carboni, Riccardo Casadei, Francesco Casella, Marco Catarci, Paolo Cerri, Damiano Chiari, Eugenio Cocozza, Giovanni Colombo, Luca Cozzaglio, Giorgio Dalmonte, Maurizio Degiuli, Maurizio De Luca, Raffaele De Luca, Nicolò De Manzini, Carlo Alberto De Pasqual, Stefano De Pascale, Nicola De Ruvo, Mariantonietta Di Cosmo, Alberto Di Leo, Massimiliano Di Paola, Amedeo Elio, Francesco Ferrara, Giovanni Ferrari, Valentino Fiscon, Uberto Fumagalli, Gianluca Garulli, Andrea Gennai, Irene Gentile, Paola Germani, Monica Gualtierotti, Francesca Guerini, Angela Gurrado, Marco Inama, Filippo La Torre, Ernesto Laterza, Pasquale Losurdo, Antonio Macrì, Alessandra Marano, Luigi Marano, Federico Marchesi, Fabio Marino, Marco Massani, Roberta Menghi, Marco Milone, Sarah Molfino, Mauro Montuori, Gianluigi Moretto, Paolo Morgagni, Emilio Morpurgo, Moukchar Abdallah, Luca Nespoli, Stefano Olmi, Raffaele Palaia, Giovanni Pallabazer, Paolo Parise, Alessandro Pasculli, Marco Pericoli Ridolfini, Antonio Pesce, Enrico Pinotti, Michele Pisano, Elia Poiasina, Vittorio Postiglione, Stefano Rausei, Antonio Rella, Fausto Rosa, Riccardo Rosati, Gianmaria Rossi, Luca Rossit, Massimo Rovatti, Laura Ruspi, Luca Sacco, Edoardo Saladino, Andrea Sansonetti, Alberto Sartori, Donatella Scaglione, Stefano Scaringi, Christian Schoenthaler, Giuseppe Sena, Michele Simone, Leonardo Solaini, Paolo Strignano, Nicola Tartaglia, Silvio Testa, Mario Testini, Guido Alberto Massimo Tiberio, Elio Treppiedi, Alessio Vagliasindi, Michele Valmasoni, Jacopo Viganò, Gianpietro Zanchettin, Andrea Zanoni, Claudio Zardini, Antonio Zerbinati

**Affiliations:** 1 General and Upper G.I. Surgery Division, University of Verona, Verona, Italy; 2 Department of Diagnostics and Public Health, University of Verona, Verona, Italy

**Keywords:** Gastric cancer, Gastrectomy, Drain, Drainage, Survey

## Abstract

Evidence against the use of prophylactic drain after gastrectomy are increasing and ERAS guidelines suggest the benefit of drain avoidance. Nevertheless, it is unclear whether this practice is still widespread. We conducted a survey among Italian surgeons through the Italian Gastric Cancer Research Group and the Polispecialistic Society of Young Surgeons, aiming to understand the current use of prophylactic drain. A 28-item questionnaire-based survey was developed to analyze the current practice and the individual opinion about the use of prophylactic drain after gastrectomy. Groups based on age, experience and unit volume were separately analyzed. Response of 104 surgeons from 73 surgical units were collected. A standardized ERAS protocol for gastrectomy was applied by 42% of the respondents. Most of the surgeons, regardless of age, experience, or unit volume, declared to routinely place one or more drain after gastrectomy. Only 2 (1.9%) and 7 surgeons (6.7%) belonging to high volume units, do not routinely place drains after total and subtotal gastrectomy, respectively. More than 60% of the participants remove the drain on postoperative day 4–6 after performing an assessment of the anastomosis integrity. Interestingly, less than half of the surgeons believe that drain is the main tool for leak management, and this percentage further drops among younger surgeons. On the other hand, drain’s role seems to be more defined for duodenal stump leak treatment, with almost 50% of the surgeons recognizing its importance. Routine use of prophylactic drain after gastrectomy is still a widespread practice even if younger surgeons are more persuaded that it could not be advantageous.

**Supplementary Information:**

The online version contains supplementary material available at 10.1007/s13304-022-01397-0.

## Introduction

Despite surgical advancement, gastric cancer surgery is still burdened by significant morbidity and mortality. A multicenter prospective database on more than one thousand gastrectomies published in 2020 reported a 30% complication rate, with a 10% and 3.5% incidence of anastomotic and duodenal stump leak, respectively [1].

Prophylactic abdominal drain is still used as a diagnostic and therapeutic tool for intrabdominal complications after gastrectomy, although evidence against its routine use is increasing. An updated meta-analysis including 3 RCTs and 7 cohort studies showed that prophylactic drain avoidance can reduce morbidity and length of stay, while not significantly affecting other major surgical outcomes [2]. Of note, these results mainly come from Eastern studies while only one was conducted in the West [3].

With the widespread adoption of Enhanced Recovery After Surgery (ERAS) protocols, surgeons are asked to reduce surgical stress by using minimally invasive approaches and avoiding unnecessary catheters and drains. Guidelines from ERAS Society strongly recommend prophylactic drain avoidance [4], however, it is unclear whether this practice is still routine or not.

We conducted a survey aiming to clarify the current practice on the use of prophylactic drain after gastrectomy in Italy, analyzing the differences between high and low volume units and young or experienced surgeons.

## Methods

### ADiGe survey group

The Abdominal drain in Gastrectomy (ADiGe) Survey group is an Italian research group established by the Italian Gastric Cancer Research Group (GIRCG) and focused on improving the knowledge about prophylactic drain use in patients undergoing gastrectomy for gastric cancer. Any surgeon performing gastrectomy for cancer in Italy is eligible to participate in the survey. Invitations to join the ADiGe Survey group were initially sent by V.M and A.V through the GIRCG and the Polispecialistic Society of Young Surgeons (SPIGC) contact list. All the other surgeons willing to join the group were evaluated with a brief interview. All the communications with the collaborators were made through the following email address: adige.group.it@gmail.com. Recruitment started in December 2019.

### Survey questionnaire

The 28 items questionnaire was formulated by V.M., J.W. and G.d.M based on the existing evidence on the utility of prophylactic drain after gastrectomy. The questionnaire was built to investigate both the current clinical practice and the standpoint on the use of prophylactic drain after gastrectomy. Answers were then related to the surgeons’ age and experience, unit volume and hospital facilities. Briefly, questions were divided into three areas of interest: surgical technique, drain management and intra and post-operative anastomotic assessment. The last part of the survey focused on each surgeon’s opinion about the role of prophylactic drain in the diagnosis and treatment of anastomotic and duodenal stump leaks. The questionnaire is fully reported in the supplementary material (Table S1).

Surgeons were divided into groups based on age and experience in gastric cancer surgery. Age groups were defined using the following ranges: “young”, between 25 and 35 years (considered as the surgical training period in Italy); “middle age” including surgeons aged between 36 and 45 and “senior” for surgeons with more than 45 years.

There is no clear definition in literature of expert gastric cancer surgeon in the West, where the incidence of gastric cancer is significantly lower than in Asian countries. A recent review article highlighted the extreme variability between 2 Western and 1 Eastern studies in defining the cut-off for a single surgeon’s volume in gastric surgery. While the USA studies identified a cut-off of 12 and 21 gastrectomies in 4 years, this raised to 35 in 3 years in the Eastern paper [5]. To set the cut-off for this study, we considered the number of gastrectomies performed each year in Italy according to the Italian PNE National Healthcare Outcomes Program [6] and the lack of a centralization policy. Based on these considerations, experience groups were defined as follow: “beginner” for surgeons that performed less than 20 gastrectomies, “intermediate” for range of 20–50 and “expert” for surgeons that already performed more than 50 gastrectomies in their career [7].

Unit volume (total number of gastrectomies performed per year) classification was based on several reports on the quality of gastric surgery and surgical volume in the West [8] and on the Italian PNE [6]. Therefore, unit volume was defined as follow: “low” (< 20 cases/year), “medium” (20–30 cases/year) and “high” (> 30 cases/year).

Questions were divided also by type of gastrectomy: only total and subtotal gastrectomy were included, as we considered that these accounted for more than 90% of all the operations performed in Europe since 2017 according to Gastrodata database [1].

The level of post-operative assistance was identified as the usual destination of a patient in the early postoperative period and included the Intensive Care Unit (ICU), the Step-Down Unit or the surgical ward. Hospital organization was further analyzed through the availability of in hospital/on call staff for surgical emergency, interventional radiology and endoscopy. Information on the preferred anastomotic technique and on intraoperative test for anastomotic integrity were asked.

The survey included specific questions on surgeons’ habit of draining total and subtotal gastrectomy, type and number of drains used and post-operative management.

At last, questions on the “perceived” utility of prophylactic drain placement in detecting and treating anastomotic and duodenal stump leakages were asked.

To try to get the best quality data for the core information we decided to keep the questionnaire as short as possible, thus some potentially interesting aspects have not been investigated (such as the correlation with minimally invasive surgery or with the type of reconstruction).

### Statistical methods

All answers were reported as frequency and percentage for categorical variables and as median and interquartile range (p25–p75) for non-normally distributed continuous variables. Statistical differences between groups (age, experience and unit volume) were evaluated using Fisher’s exact test for categorical variables and non-parametric Kruskal–Wallis test for quantitative variables.

Statistical analysis was performed using Stata^®^ software 16.1 (StataCorp LP, College Station, Texas, USA) and a *p*-value < 0.05 was considered statistically significant.

## Results

104 surgeons from 73 surgical units mailed back the complete survey. Most of the surgeons who did not join the survey reported that gastrectomies were not in their field of expertise. Among the responders 12 were young surgeons (11%) while 31 and 61 were included in the middle age (30%) and senior group (59%), respectively.

Half of the responders were considered as “expert” in performing a gastrectomy (51%) while intermediate (21%) and low experienced (28%) surgeons were equally divided. When considering unit volume, 36 surgeons came from a low volume unit (35%), 27 from a medium volume unit (26%) while 41 from a high-volume unit (39%).

Fifty-eight surgeons (56%) declared that they routinely evaluated the anastomosis integrity (e.g., pneumatic test, methylene blue test) during the operation. Nevertheless, the possibility to perform an intraoperative assessment of anastomosis viability using indocyanine green was still limited, with only 42% of the respondent who answered yes.

Surgeon for gastroesophageal emergencies and interventional endoscopist were available on call 24/7 in most of the hospitals involved in this survey (85% and 90%, respectively). On the other hand, the interventional radiology service was available 24/7 only in 66% of the hospitals, being limited to the weekdays (Mon–Fri) in 16.5% and not available at all in 13.5%.

After gastrectomy, patients were usually destined to surgical ward (58%) or step-down unit (17%), but still a quarter of the respondents preferred to monitor the patients in ICU in the early postoperative period. Interestingly, only 42% of the surgeons had access to an ERAS program for gastrectomy in their unit.

While the availability of endo-vac therapy for leaks after gastrectomy was still limited (42%), nearly all the surgical units had the possibility to use parenteral nutrition (100%), endoscopic clips (96%) or stents (95%), and image-guided percutaneous drain placement (97%).

All the features of surgeons and centers involved in the survey are reported in Table 1.

**Table 1:** Characteristics of the surgeons and hospitals participating in the survey

	Total (104)
*Age*	
25–35	12 (11)
36–45	31 (30)
> 45	61 (59)
*Experience of the surgeon*	
< 20	29 (28)
20–50	22 (21)
> 50	53 (51)
*Volume of the Hospital*	
< 20	36 (35)
20–30	27 (26)
> 30	41 (39)
*On call doctor for gastroesophageal emergencies*	
None	11 (11)
Weekdays: Daytime/24 h	5 (4)/0
Every day: Daytime/24 h	0/88 (85)
*On call doctor for interventional radiology*	
None	14 (13.5)
Weekdays: Daytime / 24 h	14 (13.5)/3 (3)
Every day: Daytime / 24 h	4 (4)/69 (66)
*On call doctor for interventional endoscopy*	
None	2 (2)
Weekdays: Daytime / 24 h	6 (6)/0
Every day: Daytime / 24 h	2 (2)/94 (90)
*Routine postoperative destination of a patient after gastrectomy*	
Ward	60 (58)
Step Down Unit	18 (17)
Intensive Care Unit	25 (24)
Other	1 (1)
*Formalized ERAS protocol for gastrectomy*	
Yes	44 (42)
*Intraoperative Indigo-Cyanine Green availability*	
Yes	44 (42)
*Routine intraoperative assessment of the anastomosis*	
Yes	58 (56)
Technique/s available to treat anastomotic leak*	
Parenteral nutrition	104 (100)
Endoscopic clips	100 (96)
Endoscopic/radiologically placed covered stent	99 (95)
EndoVac/endosponge therapy	44 (42)
Interventional guided drainage	101 (97)

### Anastomotic technique and intraoperative assessments

The preferred technique for esophago-jejunal anastomosis after total gastrectomy was the circular stapled, chosen by over 80% of the surgeons. A larger variability emerged in the gastro-jejunal anastomosis with medium (70%) and high volume (54%) units that favored the stapled side-to-side while low volume units mainly adopted the handsewn (47%). Half of the units had access to indocyanine green imaging of the anastomosis, irrespective of unit volume. Interestingly, routine intraoperative assessment of the anastomosis integrity decreased along with the increase in units’ experience (*p* = 0.009). All details on surgical technique preferences analyzed according to age and experience of the surgeon and to the unit volume are reported in Table 2.

**Table 2:** Anastomotic technique and intraoperative assessments according to surgeon’s age, experience and unit volume

	Tot	Age				Experience				Volume			
	*n* = 104	25–35 *n* = 12	36–45 *n* = 31	> 45 *n* = 6	*p* value	< 20 *n* = 29	20–50 *n* = 22	> 50 *n* = 53	*p* value	< 20 *n* = 36	20–30 *n* = 27	> 30 *n* = 41	*p* value
*Preferred technique for esophago-jejunal anastomosis*					0.591				0.346				0.881
Handsewn	4 (4)	1 (8)	2 (7)	1 (2)		2 (7)	1 (5)	1 (2)		1 (3)	1 (4)	2 (5)	
Circular Stapled	90 (86)	11 (92)	28 (90)	51 (83)		26 (90)	17 (77)	47 (88)		32 (89)	22 (81)	36 (88)	
OrVil™	1 (1)	0	0	1 (2)		0	1 (4)	0		0	1 (4)	0	
Stapled side-to-side with suturing (Orringer style)	7 (7)	0	1 (3)	6 (10)		1 (3)	3 (14)	3 (6)		3 (8)	2 (7)	2 (5)	
Other	2 (2)	0	0	2 (3)		0	0	2 (4)		0	1 (4)	1 (2)	
*Preferred technique for gastro-jejunal anastomosis*					0.053				0.279				**0.004**
Handsewn	34 (33)	8 (67)	6 (19)	20 (33)		14 (48)	6 (27)	14 (26)		17 (47)	5 (19)	12 (29)	
Circular Stapled	17 (16)	0	8 (26)	9 (15)		5 (17)	1 (5)	11 (21)		8 (22)	3 (11)	6 (15)	
OrVil™	1(1)	0	0	1 (2)		0	0	1 (2)		0	0	1 (2)	
Stapled side-to-side with suturing (Orringer style)	49 (47)	3 (25)	16 (52)	30 (49)		9 (31)	14 (64)	26 (49)		8 (22)	19 (70)	22 (54)	
Other	3 (3)	1 (8)	1 (3)	1 (2)		1 (4)	1 (4)	1 (2)		3 (9)	0	0	
*Intraoperative Indigo-Cyanine Green availability*					0.132				0.078				0.247
Yes	58 (56)	6 (50)	13 (42)	39 (64)		11 (38)	14 (64)	33 (62)		16 (44)	17 (63)	25 (61)	
No	46 (44)	6 (50)	18 (58)	22 (36)		18 (62)	8 (36)	20 (38)		20 (56)	10 (37)	16 (39)	
*Routine intraoperative assessment of the anastomosis*					0.144				0.195				**0.009**
Yes	62 (60)	5 (42)	16 (52)	41 (67)		14 (48)	12 (54)	36 (68)		27 (75)	18 (67)	17 (41)	
No	42 (40)	7 (58)	15 (48)	20 (33)		15 (52)	10 (46)	17 (32)		9 (25)	9 (33)	24 (59)	

Data are reported as number (percentage)

Significant values are highlighted in bold

### Drain management

As reported in Table 3, after *total gastrectomy* all but two of the participants routinely place one or more abdominal drain. The majority of surgeons place 2 drains (59%), but a trend towards a reduction in the number of drains used is apparent among high volume units (*p* = 0.065).

**Table 3:** Drain habits and post-operative management according to surgeon’s age, experience and unit volume

	Tot	Age				Experience				Volume			
	*n* = 104	25–35 *n* = 12	36–45 *n* = 31	> 45 *n* = 6	*p* value	< 20 *n* = 29	20–50 *n* = 22	> 50 *n* = 53	*p* value	< 20 *n* = 36	20–30 *n* = 27	> 30 *n* = 41	*p* value
*Prophylactic drain/s placement in TG*					0.647				0.713				0.336
Yes	102 (98)	12(100)	31(100)	59 (97)		29(100)	22 (100)	51 (96)		36(100)	27 (100)	39 (95)	
No	2 (2)	0	0	2 (3)		0	0	2 (4)		0	0	2 (5)	
*If yes, number of drains used*					0.794				0.164				0.065
1	36 (35)	5 (42)	12 (39)	19 (32)		14 (48)	4 (18)	18 (35)		9 (25)	9 (33)	18 (46)	
2	60 (59)	6 (50)	17 (55)	37 (63)		13 (45)	16 (73)	31 (61)		22 (61)	17 (63)	21 (54)	
> 2	6 (6)	1 (8)	2 (6)	3 (5)		2 (7)	2 (9)	2 (4)		5 (14)	1 (4)	0	
*Prophylactic drain/s placement in STG*					0.062				0.134				**0.003**
Yes	97 (93)	12(100)	26 (84)	59 (97)		29(100)	21 (95)	47 (89)		36(100)	27 (100)	34 (83)	
No	7 (7)	0	5 (16)	2 (3)		0	1 (5)	6 (11)		0	0	7 (17)	
*If yes, number of drains used*					0.716				0.525				**0.007**
1	64 (66)	9 (75)	17 (65)	38 (64)		19 (66)	11 (52)	34 (72)		18 (50)	17 (63)	29 (85)	
2	29 (30)	2 (17)	8 (31)	19 (32)		9 (31)	9 (43)	11 (23)		14 (39)	10 (37)	5 (15)	
> 2	4 (4)	1 (8)	1 (4)	2 (4)		1 (3)	1 (5)	2 (5)		4 (11)	0	0	
*Drain/s location*^a^													
Perianastomotic	93 (91)	12(100)	29 (93)	52 (88)	0.609	27 (93)	21 (95)	45 (88)	0.729	34 (94)	24 (89)	35 (90)	0.744
Duodenal stump	81 (79)	9 (75)	22 (71)	50 (85)	0.264	22 (76)	18 (82)	41 (80)	0.854	32 (89)	22 (81)	27 (69)	0.116
Other	8 (8)	1 (8)	4 (13)	3 (5)	0.357	3 (10)	2 (9)	3 (6)	0.706	5 (14)	2 (7)	1 (3)	0.211
*Type of drain used*					0.623				0.314				0.219
Open	35 (34)	6 (50)	12 (39)	17 (29)		11 (38)	7 (32)	17 (33)		14 (39)	6 (22)	15 (38)	
close passive	50 (49)	5 (42)	15 (48)	30 (51)		11 (38)	10 (45)	29 (57)		19 (53)	13 (48)	18 (46)	
close active	17 (17)	1 (8)	4 (13)	12 (20)		7 (24)	5 (23)	5 (10)		3 (8)	8 (30)	6 (16)	
*POD drain removal, median (IQR)*					0.225				0.252				0.326
≤ 3 days	21 (21)	2 (17)	8 (26)	11 (19)		8 (28)	1 (5)	12 (23)		7 (19)	5 (19)	9 (23)	
4–6 days	66 (65)	8 (66)	22 (71)	36 (61)		18 (62)	17 (77)	31 (61)		20 (56)	19 (70)	27 (69)	
> 6 days	15 (14)	2 (17)	1 (3)	12 (20)		3 (10)	4 (18)	8 (16)		9 (25)	3 (11)	3 (8)	

Data are reported as number (percentage)

Significant values are highlighted in bold

*POD* postoperative day, *IQR* interquartile range, *TG* total gastrectomy, *STG* subtotal gastrectomy

^a^Mark all that apply

Abdominal drain is still widely used also in *subtotal gastrectomy*. Nevertheless 7 surgeons (17%) belonging to high volume units, do not routinely place any drain (*p* = 0.003). More than half of the surgeons use only one drain after subtotal gastrectomy; however, a significant difference is apparent when considering unit volume: 50% in low volume, 63% in medium volume and 85% in high volume (*p* = 0.007) (Table 3).

Of note, all the surgeons that do not routinely place a prophylactic drain belong to high volume units with a standardized ERAS pathway and a 24-h availability of interventional radiology and endoscopy (Table S2).

As expected, drains are usually placed close to the anastomosis and to the duodenal stump, while some surgeons declared to place a further drain in the pouch of Douglas.

A large variability in the type of drain used emerged, and the choice is probably related to surgical habit and favors open or close passive systems. Most surgeons (65%) leave the drain in place for 4–6 days, with no significant difference related to age, experience or unit volume.

### Anastomosis and leak management

More than half of the surgeons that declared to place a prophylactic drain routinely perform a postoperative assessment of the anastomosis before drain removal. When a postoperative examination is planned, for the most part, both total and subtotal gastrectomy are tested on postoperative day 5 (range: 1–9). When anastomotic leak is suspected, water soluble contrast swallow is the preferred technique (71%), while endoscopy is seldom used (6%). Within the treatment strategies for surgical complications, Endoscopic clip and stent and EUS/CT guided drainage of collections are available in most units, while endoscopic vacuum therapy use is a prerogative of the high-volume units (Table 4).

**Table 4:** Anastomosis and leak management according to surgeon’s age, experience and unit volume

	Tot	Age				Experience				Volume			
	n = 104	25–35 *n* = 12	36–45 *n* = 31	> 45 *n* = 6	*p* value	< 20 *n* = 29	20–50 *n* = 22	> 50 *n* = 53	*p* value	< 20 *n* = 36	20–30 *n* = 27	> 30 *n* = 41	*p* value
*Routine postoperative assessment of the anastomosis*					0.274				**0.018**				0.901
Yes	64 (63)	10 (83)	17 (57)	37 (63)		19 (65)	19 (86)	26 (52)		23 (64)	18 (67)	23 (60)	
No	38 (37)	2 (17)	13 (43)	22 (37)		10 (35)	3 (14)	24 (48)		13 (36)	9 (33)	15 (40)	
*If yes, assessment performed only in TG*					0.725				0.596				0.352
Yes	11 (17)	1 (10)	4 (23)	6 (16)		3 (16)	2 (10)	6 (23)		2 (9)	3 (17)	6 (26)	
No	53 (83)	9 (90)	13 (77)	31 (84)		16 (84)	17 (90)	20 (77)		21 (91)	15 (83)	17 (74)	
*POD assessment of the anastomosis, median (IQR)*	5 (4.5–7)	6 (4–7)	5 (4–5)	5 (5–7)	0.239	5 (4–7)	5 (5–7)	5 (5–6)	0.802	5 (4–7)	5 (4–6)	5 (5–7)	0.350
*Exam/s used for postoperative assessment*^a^													
Barium/Water soluble contrast swallow	74 (71)	10 (83)	18 (58)	46 (75)	0.155	20 (69)	17 (77)	37 (70)	0.812	24 (67)	21 (78)	29 (71)	0.628
Computed Tomography	14 (13)	1 (8)	3 (10)	10 (16)	0.639	4 (14)	1 (5)	9 (17)	0.419	6 (17)	3 (11)	5 (12)	0.817
Endoscopy	6 (6)	0	2 (6)	4 (7)	1.00	0	0	6 (11)	**0.046**	2 (6)	0	4 (10)	0.236
Other	13 (12)	2 (17)	4 (13)	7 (11)	0.833	5 (17)	4 (18)	4 (7)	0.280	6 (17)	5 (18)	2 (5)	0.133
Technique/s available to treat anastomotic leak*													
Parenteral Nutrition	104(100)	12(100)	31(100)	61 (100)	–	29(100)	22 (100)	53(100)	–	36(100)	27 (100)	41(100)	–
Endoscopic clips	100 (96)	10 (83)	31(100)	59 (97)	0.056	27 (93)	22 (100)	51 (96)	0.661	33 (92)	26 (96)	41(100)	0.130
Endoscopic/radiologically placed covered stent	99 (95)	12(100)	29 (93)	58 (95)	1.00	27 (93)	21 (95)	51 (96)	0.837	33 (92)	26 (96)	40 (98)	0.524
EndoVac/Endosponge therapy	44 (42)	4 (33)	12 (39)	28 (46)	0.679	8 (28)	11 (50)	25 (47)	0.161	13 (36)	7 (26)	24 (58)	**0.019**
Interventional guided drainage of collections	101 (97)	12(100)	30 (97)	59 (97)	1.00	27 (93)	22 (100)	52 (98)	0.300	34 (94)	26 (96)	41(100)	0.356

Data are reported as number (percentage) or median (interquartile range)

Significant values are highlighted in bold

*POD* postoperative day, *IQR* interquartile range, *TG* total gastrectomy

^a^mark all that apply

### Role of abdominal drain in anastomotic and duodenal leaks

**Fig. 1:**
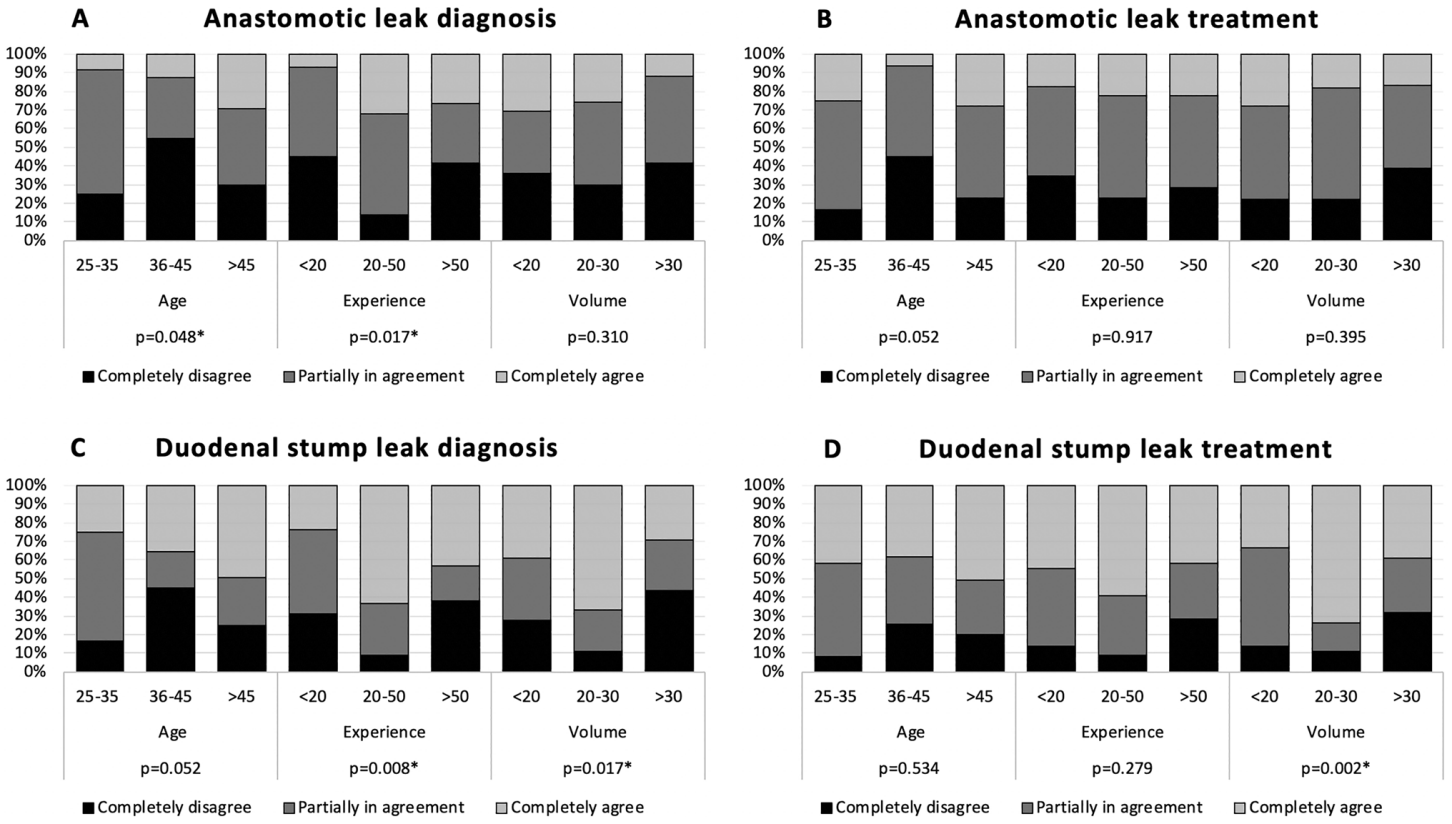
Perceived utility of the prophylactic drain placement in detecting and treating anastomotic (**A** and **B**) and duodenal (**C** and **D**) stump leakages by surgeon’s age, experience, and unit volume

A large variability among surgeons was apparent when the role of prophylactic drain on anastomotic leak *diagnosis* and *treatment* was investigated (Fig. 1A and B). While 22% of the respondents consider the prophylactic drain as the main tool for leak *diagnosis*, it seems that younger (8%) and less experienced surgeons (7%) are less keen to believe in drain’s key function. When drain role in anastomotic leak *treatment* is investigated, this difference is no longer observable, and overall, while 50% of the surgeons are partially in agreement, the others are almost equally divided between completely disagree (29%) and completely agree (21%).

When duodenal stump leak is investigated, a higher percentage of surgeons believe in prophylactic drain role for both *diagnosis* (42%) and *treatment* (46%) (Fig. 1C and D). Consistently with what observed in the anastomotic leak *diagnosis* question, younger (25%) and less experienced surgeons (24%) are more skeptic in prophylactic drain’s role for duodenal stump leak *diagnosis* compared with the other groups. Interestingly, surgeons belonging to high volume units are significantly less keen to believe in the diagnostic role of prophylactic drain for duodenal stump leak (*p* = 0.017). Answers to the duodenal leak *treatment* question evidenced that almost half (46%) of surgeons agreed with the role of prophylactic drain with no significant difference among age and experience groups. Surprisingly, most of the medium volume surgeons (74%) consider the drain as the main tool to treat a duodenal leak compared with 33% of the low volume and 39% of the high-volume surgeons (*p* = 0.002). Data are fully reported in supplementary material (Table S3).

**Fig. 2:**
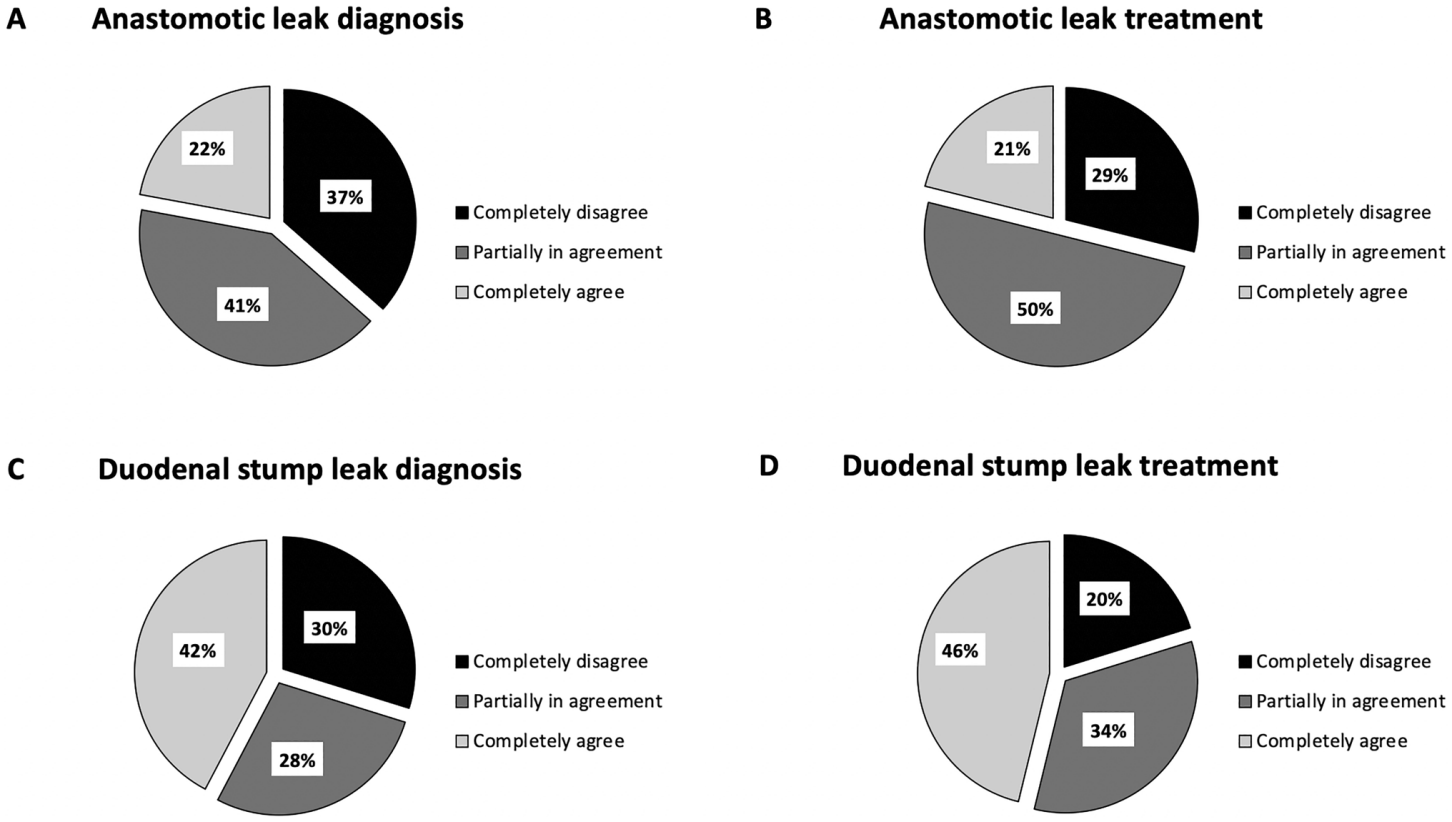
Perceived utility of the prophylactic drain placement in detecting and treating anastomotic and duodenal stump leakages only by surgeons that routinely placed at least one abdominal drain after gastrectomy

The perceived utility of the prophylactic drain in detecting and treating anastomotic and duodenal stump leakages among the surgeons that routinely placed at least one drain is represented in Fig. 2. As expected, none of the surgeons that do not place any prophylactic drain consider it as the main tool for diagnosis or treatment of anastomotic and duodenal stump leaks.

## Discussion

This study evidenced how routine use of one or more prophylactic drain after gastrectomy is still a widespread practice among Italian surgeons regardless of age, experience or unit volume. The large cohort analyzed shows how Italian surgeons performing gastrectomy have a 24/7 surgeon and endoscopist in most cases, while the interventional radiology is often limited to the weekdays. Moreover, despite an established ERAS program is still lacking, patients are treated in the surgical ward or in a step-down unit in 75% of cases, thus reducing the ICU-related complications. Interestingly, the use of indocyanine green to assess the anastomosis viability (42%) and the endo-vac therapy to treat the leakage (42%) are still limited to some centers and this could have an impact on the decision to place a prophylactic drain.

Only two surgeons (1.9%) declared not to routine insert a drain after total gastrectomy while the number increased to seven (6.7%) for subtotal gastrectomy. As expected, this “no drain” group of surgeons belongs to high volume units with a standardized ERAS pathway and a 24-h availability of interventional radiology and endoscopy.

Interestingly, less than half of the surgeons believe that prophylactic drain is the main tool for leak management, and this percentage further drops among younger surgeons. This result raises a question on how this practice is related to habit rather than to a real convincement. The role of prophylactic drain seems to be more defined for duodenal stump leak treatment, with almost 50% of the surgeons recognizing its importance.

The debate on the utility of prophylactic abdominal drain was already present in the early twentieth century in a paper by Yates [9]. Over 100 years later, despite the growing evidence against routine drain placement in many surgical specialties [10–13] and ERAS guidelines recommendations [4, 14–17], the discussion is still open. Upper gastrointestinal surgery is a stronghold of practice such as drain, nasogastric tube and prolonged fasting, but in recent years evidence against their use are increasing. Concerning prophylactic drain after gastrectomy, ERAS guidelines from 2014 strongly recommended with a high level of evidence to avoid its routinary placement [4]. Moreover, an updated meta-analysis including 3 RCTs and 7 cohort studies showed that prophylactic drain avoidance can reduce morbidity and length of stay, while not significantly affecting other major surgical outcomes [2]. Of note, the included studies came mainly from Eastern countries and therefore this could blunder the impact on Western surgical practice. A recent retrospective study from Korea confirmed that prophylactic drain insertion did not reduce the incidence of intra-abdominal complications and gives no significant advantage in their early diagnosis and management [18].

Nevertheless, clinical practice and evidence are not always straightforward, and this has already been discussed in two articles on the use of prophylactic drain after kidney transplantation and breast surgery [19, 20]. The first article highlighted how, despite a paucity of evidence, more than 60% of the participants reported routine drain insertion, describing habit and concern about bleeding as the main reasons for this practice [19]. Similar results have been reported in a survey among breast surgeons from United Kingdom and Ireland, with the majority (71.5%) of the respondents reporting to regularly insert a drain after bilateral breast reduction surgery [20] despite the evidence published against this practice [21]. Interestingly, drain use was significantly reduced in high volume units.

The result of this survey indicates that, in Italy, prophylactic drain use in gastric cancer surgery is still widespread, with more than 90% of the participants that routinely use of at least one drain. It’s worth noting that surgeons are more concerned about duodenal stump than anastomotic leak. Indeed, nearly half of them consider prophylactic drain as the main tool for diagnosis and treatment of duodenal stump leak and this persuasion becomes more apparent in experienced and low volume surgeons. Despite results from literature evidenced the efficacy of conservative treatment for duodenal stump leak, leading to a resolution in more than 90% of cases [22], it must be noted that it included both medical/nutritional therapy as well as percutaneous drain placement. This leads to a difficult analysis of the efficacy of prophylactic drain in diagnosis and treatment of this complication. It is, therefore, possible that low volume units, often with fewer facilities, rely more in prophylactic drain as diagnostic and therapeutic tool for duodenal stump leak compared with high volume centers.

This survey highlighted how less than 50% of the surgeons have access to an ERAS program for gastrectomy in their center. Notably, this result is in line with a recent nationwide survey from Korea that reported a 50.6% application of ERAS among the participant surgeons [23]. Moreover, the same study indicates that nearly 70% of the surgeons routinely placed a prophylactic drain after gastrectomy, suggesting that drain use is still widespread not only in Italy but also worldwide.

Concluding, the results of this survey evidenced that prophylactic drain after gastrectomy is still widely used in Italy, even if many surgeons are persuaded that it could not be advantageous. Of note, younger surgeons seem less keen on believing in this practice. A possible explanation for this reticence could be the limited evidence coming from Western studies and the low prevalence of established ERAS programs.

We think that new and stronger evidence are needed to define the role of prophylactic drain after gastrectomy. We expect that the ongoing multicenter randomized ADiGe Trial will provide further evidence in this regard, thus guiding the future clinical practice and ERAS guidelines for gastric cancer [24].

## Supplementary Information

Below is the link to the electronic supplementary material.

Supplementary file1 (DOCX 33 KB)
