# Supplementary material for: Current practice on the use of prophylactic drain after gastrectomy in Italy: the Abdominal Drain in Gastrectomy (ADiGe) survey
Source: Updates Surg. 2022 Oct 24;74(6):1839–49. doi: 10.1007/s13304-022-01397-0 (PMC9674733; doi:10.1007/s13304-022-01397-0)
Supplement: Supplementary file 1 — Supplementary file1 (DOCX 33 KB) [file 13304_2022_1397_MOESM1_ESM.docx]

**Supplementary materials**

**Table S1**

28-items questionnaire.

| Age | 1. 25-35 2. 36-45 3. >45 |
| --- | --- |
| Number of gastrectomies performed | 1. <20 2. 20-50 3. >50 |
| Number of gastrectomies performed in your unit in 2018 (01/01/2018-31/12/18)  *please record the total number of procedures performed by all surgeons in your unit | (1) <20  (2) 20-30  (3) >30 |
| Is your unit equipped with a 24-hour consultant/attending on call doctor for gastroesophageal emergencies? | 1. None 2. Weekdays Daytime e.g. 08:00-17:00 3. Weekdays 24 hours 4. Every day Daytime e.g. 08:00-17:00 5. Every day 24 hours |
| Is your hospital equipped with a 24-hour on call doctor for interventional radiology? | 1. None 2. Weekdays Daytime e.g. 08:00-17:00 3. Weekdays 24 hours 4. Every day Daytime e.g. 08:00-17:00 5. Every day 24 hours |
| Is your hospital equipped with a 24-hour on call doctor for interventional endoscopy? | 1. None 2. Weekdays Daytime e.g. 08:00-17:00 3. Weekdays 24 hours 4. Every day Daytime e.g. 08:00-17:00 5. Every day 24 hours |
| Which is the routine postoperative destination of a patient after gastrectomy?  *Step Down Units (level 2 care) are an intermediary level of care between a general surgical ward and intensive care units. | 1. Ward 2. Step Down Unit* 3. Intensive Care Unit 4. Other |
| Does your unit have a formalized ERAS (Enhanced Recovery After Surgery) protocol for gastrectomy? | 1. Yes 2. No |
| Which is your preferred technique to perform the esophago-jejunal anastomosis? | 1. Handsewn 2. Circular Stapled 3. OrVil™ 4. Stapled side-to-side with suturing (Orringer style) 5. Other 6. Not Applicable- do not perform |
| Which is your preferred technique to perform the gastro-jejunal anastomosis? | 1. Handsewn 2. Circular Stapled 3. OrVil™ 4. Stapled side-to-side with suturing (Orringer style) 5. Other 6. Not Applicable- do not perform |
| Does you unit have access to Indigo-Cyanine Green intraoperative assessment? | 1. Yes 2. No |
| Does your unit perform a routine (even if no concerns) intra-operative assessment of the anastomosis (Blue-Test or Pneumatic Test)? | 1. Yes 2. No |
| Do you routinely place at least one prophylactic abdominal drain in total gastrectomy? | 1. Yes 2. No |
| If yes, how many drains do you routinely use? | (1) 1  (2) 2  (3) > 2 |
| Do you routinely place at least one prophylactic abdominal drain in subtotal gastrectomy? | 1. Yes 2. No |
| If yes, how many drains do you routinely use? | (1) 1  (2) 2   1. > 2 |
| Where do you usually place the drain/s?  * Please mark all that apply | 1. perinastomotic 2. duodenal stump 3. other (specify) |
| Which type of drain do you routinely use? | 1. open 2. close passive 3. close active |
| How many days do you leave in place the prophylactic abdominal drain if no complication occurs? | 1. <= 3 days 2. 4-6 days 3. > 6 days |
| Do you routinely perform a post-operative assessment of the anastomosis before drain is removed? | 1. Yes 2. No |
| If yes, do you routinely perform the assessment only in total gastrectomy? | 1. Yes 2. No |
| When is the assessment performed?  *please indicate the postoperative day |  |
| Which exam do you usually use to assess the anastomosis?  * Please mark all that apply | 1. Barium/Water soluble contrast swallow 2. Computed Tomography (CT) 3. Endoscopy 4. Other |
| Which of the following techniques for anastomotic leak treatment are available in your unit?  * Please mark all that apply | 1. Parenteral Nutrition 2. Endoscopic clips 3. Endoscopic/radiologically placed covered stent 4. EndoVac/Endosponge therapy 5. Interventional guided drainage of abdominal/thoracic collections |
| Do you think that prophylactic drain is the main tool for **anastomotic leak diagnosis**?  (0=completely disagree; 5=completely agree) | 0 – 1 – 2 – 3 – 4 - 5 |
| Do you think that prophylactic drain is the main tool for **anastomotic leak treatment**?  (0=completely disagree; 5=completely agree) | 0 – 1 – 2 – 3 – 4 - 5 |
| Do you think that prophylactic drain is the main tool for **duodenal stump leak diagnosis**?  (0=completely disagree; 5=completely agree) | 0 – 1 – 2 – 3 – 4 - 5 |
| Do you think that prophylactic drain is the main tool for **duodenal stump leak treatment**?  (0=completely disagree; 5=completely agree) | 0 – 1 – 2 – 3 – 4 - 5 |

**Table S2**

Comparison of hospital facilities between “drain” and “no drain” surgeons for total and subtotal gastrectomy. Data are reported as number (percentage).

|  | **Tot** | **Drain in total gastrectomy** | | | **Drain in subtotal gastrectomy** | | |
| --- | --- | --- | --- | --- | --- | --- | --- |
|  | **n = 104** | **Yes**  **n=102** | **No**  **n=2** | **p value** | **Yes**  **n=97** | **No**  **n=7** | **p value** |
| *24-hour on call doctor for gastroesophageal emergencies* |  |  |  | 1.000 |  |  | 1.000 |
| None | 11 (11) | 11 (11) | 0 |  | 11 (11) | 0 |  |
| Weekdays Daytime e.g. 08:00-17:00 | 5 (4) | 5 (5) | 0 |  | 5 (5) | 0 |  |
| Every day 24 hours | 88 (85) | 86 (84) | 2 (100) |  | 81 (84) | 7 (100) |  |
| *24-hour on call doctor for interventional radiology* |  |  |  | 1.000 |  |  | 0.650 |
| None | 14 (13.5) | 14 (13.7) | 0 |  | 14 (14) | 0 |  |
| Weekdays Daytime e.g. 08:00-17:00 | 14 (13.5) | 14 (13.7) | 0 |  | 14 (14) | 0 |  |
| Weekdays 24 hours | 3 (3) | 3 (3) | 0 |  | 3 (3) | 0 |  |
| Every day Daytime e.g. 08:00-17:00 | 4 (4) | 4 (4) | 0 |  | 4 (4) | 0 |  |
| Every day 24 hours | 69 (66) | 67 (65.7) | 2 (100) |  | 62 (65) | 7 (100) |  |
| *24-hour on call doctor for interventional endoscopy* |  |  |  | 1.000 |  |  | 1.000 |
| None | 2 (2) | 2 (2) | 0 |  | 2 (2) | 0 |  |
| Weekdays Daytime e.g. 08:00-17:00 | 6 (6) | 6 (6) | 0 |  | 6 (6) | 0 |  |
| Every day Daytime e.g. 08:00-17:00 | 2 (2) | 2 (2) | 0 |  | 2 (2) | 0 |  |
| Every day 24 hours | 94 (90) | 92 (90) | 2 (100) |  | 87 (90) | 7 (100) |  |
| *Routine postoperative destination of a patient after gastrectomy* |  |  |  | 1.000 |  |  | **0.006** |
| Ward | 60 (58) | 58 (57) | 2 (100) |  | 58 (60) | 2 (29) |  |
| Step Down Unit | 18 (17) | 18 (18) | 0 |  | 13 (13) | 5 (71) |  |
| Intensive Care Unit (ICU) | 25 (24) | 25 (24) | 0 |  | 25 (26) | 0 |  |
| Other | 1 (1) | 1 (1) | 0 |  | 1 (1) | 0 |  |
| *Formalized ERAS protocol for gastrectomy* |  |  |  | 0.177 |  |  | **0.002** |
| Yes | 44 (42) | 42 (41) | 2 (100) |  | 37 (38) | 7 (100) |  |
| No | 60 (58) | 60 (59) | 0 |  | 60 (62) | 0 |  |
| *Intraoperative Indigo-Cyanine Green availability* |  |  |  | 0.502 |  |  | 0.697 |
| Yes | 44 (42) | 56 (55) | 2 (100) |  | 55 (57) | 3 (43) |  |
| No | 60 (58) | 46 (45) | 0 |  | 42 (43) | 4 (57) |  |
| *Routine intraoperative assessment of the anastomosis* |  |  |  | 0.514 |  |  | 1.000 |
| Yes | 58 (56) | 60 (59) | 2 (100) |  | 58 (60) | 4 (57) |  |
| No | 46 (44) | 42 (41) | 0 |  | 39 (40) | 3 (43) |  |
| *Technique/s available to treat anastomotic leak** |  |  |  |  |  |  |  |
| Parenteral Nutrition | 104 (100) | 102 (100) | 2 (100) | --- | 97 (100) | 7 (100) | --- |
| Endoscopic clips | 100 (96) | 98 (96) | 2 (100) | 1.00 | 93 (96) | 7 (100) | 1.00 |
| Endoscopic/radiologically placed covered stent | 99 (95) | 97 (95) | 2 (100) | 1.00 | 92 (95) | 7 (100) | 1.00 |
| EndoVac/Endosponge therapy | 44 (42) | 42 (41) | 2 (100) | 0.177 | 37 (38) | 7 (100) | **0.002** |
| Interventional guided drainage of abdominal/thoracic collections | 101 (97) | 99 (97) | 2 (100) | 1.00 | 94 (97) | 7 (100) | 1.00 |

***** mark all that apply

Significant values are highlighted in bold

**Table S3**

Perceived role of abdominal drain in anastomotic and duodenal leaks diagnosis and treatment, according to surgeon’s age, experience and unit volume. Data are reported as number (percentage).

|  | **Tot** | **Age** | | | | **Experience** | | | | **Volume** | | | |
| --- | --- | --- | --- | --- | --- | --- | --- | --- | --- | --- | --- | --- | --- |
|  | n=104 | 25-35 n=12 | 36-45 n=31 | >45  n=61 | p value | <20 n=29 | 20-50 n=22 | >50 n=53 | p value | <20 n=36 | 20-30 n=27 | >30 n=41 | p value |
| *Drain is the main tool for anastomotic leak diagnosis* |  |  |  |  | **0.048** |  |  |  | **0.017** |  |  |  | 0.310 |
| completely disagree | 38 (37) | 3 (25) | 17 (55) | 18 (30) |  | 13 (45) | 3 (13) | 22 (42) |  | 13 (36) | 8 (30) | 17 (42) |  |
| partially in agreement | 43 (41) | 8 (67) | 10 (32) | 25 (40) |  | 14 (48) | 12 (55) | 17 (32) |  | 12 (33) | 12 (44) | 19 (46) |  |
| completely agree | 23 (22) | 1 (8) | 4 (13) | 18 (30) |  | 2 (7) | 7 (32) | 14 (26) |  | 11 (31) | 7 (26) | 5 (12) |  |
| *Drain is the main tool for anastomotic leak treatment* |  |  |  |  | 0.052 |  |  |  | 0.917 |  |  |  | 0.395 |
| completely disagree | 30 (29) | 2 (17) | 14 (45) | 14 (23) |  | 10 (35) | 5 (23) | 15 (28) |  | 8 (22) | 6 (22) | 16 (39) |  |
| partially in agreement | 52 (50) | 7 (58) | 15 (49) | 30 (49) |  | 14 (48) | 12 (54) | 26 (49) |  | 18 (50) | 16 (59) | 18 (44) |  |
| completely agree | 22 (21) | 3 (25) | 2 (6) | 17 (28) |  | 5 (17) | 5 (23) | 12 (23) |  | 10 (28) | 5 (19) | 7 (17) |  |
| *Drain is the main tool for duodenal stump leak diagnosis* |  |  |  |  | 0.052 |  |  |  | **0.008** |  |  |  | **0.017** |
| completely disagree | 31 (30) | 2 (16.7) | 14 (45) | 15 (25) |  | 9 (31) | 2 (9) | 20 (38) |  | 10 (28) | 3 (11) | 18 (44) |  |
| partially in agreement | 29 (28) | 7 (58.3) | 6 (19) | 16 (26) |  | 13 (45) | 6 (27) | 10 (19) |  | 12 (33) | 6 (22) | 11 (27) |  |
| completely agree | 44 (42) | 3 (25.0) | 11 (36) | 30 (49) |  | 7 (24) | 14 (64) | 23 (43) |  | 14 (39) | 18 (67) | 12 (29) |  |
| *Drain is the main tool for duodenal stump leak treatment* |  |  |  |  | 0.534 |  |  |  | 0.279 |  |  |  | **0.002** |
| completely disagree | 21 (20) | 1 (8) | 8 (26) | 12 (20) |  | 4 (14) | 2 (9) | 15 (28) |  | 5 (14) | 3 (11) | 13 (32) |  |
| partially in agreement | 35 (34) | 6 (50) | 11 (35) | 18 (30) |  | 12 (41) | 7 (32) | 16 (30) |  | 19 (53) | 4 (15) | 12 (29) |  |
| completely agree | 48 (46) | 5 (42) | 12 (39) | 31 (50) |  | 13 (45) | 13 (59) | 22 (42) |  | 12 (33) | 20 (74) | 16 (39) |  |

Significant values are highlighted in bold
